# Double trouble: incidental finding of a duplicated ureter using intraureteral indocyanine green during laparoscopic colorectal surgery

**DOI:** 10.1093/jscr/rjag046

**Published:** 2026-02-08

**Authors:** Jessica Falon, Fahaad Ahmed, Sanjna Vijayakumar, Edgardo Solis, Joseph Do Woong Choi, James Wei Tatt Toh

**Affiliations:** Faculty of Medicine and Health, University of Sydney, Science Rd, Camperdown NSW 2050, Australia; Department of Colorectal Surgery, Westmead Hospital, Cnr Hawkesbury Road and Darcy Road, Westmead NSW 2145, Australia; Faculty of Medicine and Health, University of Sydney, Science Rd, Camperdown NSW 2050, Australia; Department of Colorectal Surgery, Westmead Hospital, Cnr Hawkesbury Road and Darcy Road, Westmead NSW 2145, Australia; Department of Colorectal Surgery, Westmead Hospital, Cnr Hawkesbury Road and Darcy Road, Westmead NSW 2145, Australia; Faculty of Medicine and Health, University of Sydney, Science Rd, Camperdown NSW 2050, Australia; Department of Colorectal Surgery, Westmead Hospital, Cnr Hawkesbury Road and Darcy Road, Westmead NSW 2145, Australia; Faculty of Medicine and Health, University of Sydney, Science Rd, Camperdown NSW 2050, Australia; Department of Colorectal Surgery, Westmead Hospital, Cnr Hawkesbury Road and Darcy Road, Westmead NSW 2145, Australia; Faculty of Medicine and Health, University of Sydney, Science Rd, Camperdown NSW 2050, Australia; Department of Colorectal Surgery, Westmead Hospital, Cnr Hawkesbury Road and Darcy Road, Westmead NSW 2145, Australia

**Keywords:** colorectal surgery, laparoscopic surgery, indocyanine green, ureteric injury

## Abstract

Ureteric injury is a feared complication in colorectal surgery. Anomalous genitourinary anatomy is common, with duplication of the ureter present in up to 6% of patients. This may increase the risk of inadvertent injury. We describe a laparoscopic anterior resection performed for recurrent sigmoid diverticulitis, where there were difficulties visualizing the left ureter. Intraureteral indocyanine green (ICG) was instilled via a temporary ureteric stent to assist with its identification. This unexpectedly revealed a previously unrecognized narrow-caliber bifid ureter, and enabled both branches of the ureter to be protected from injury. The ureteric stent was removed at completion of the case. The patient experienced transient hematuria post-stent removal, with no other adverse events. Intraureteral ICG is a safe and effective method for intraoperative identification of the ureter, and should be considered particularly in cases where unfavorable anatomy or dense inflammatory adhesions prevent adequate visualization and safe dissection.

## Introduction

Identification and protection of the ureters in colorectal surgery is an important step in preventing ureteric injury. Once the ureters are identified, the tempo of surgery typically increases as the danger of ureteric injury is thought to have been ameliorated. However, even with careful visual identification, anatomic anomalies exist such as duplicated ureters. Meticulous dissection must therefore be maintained, as injury to the ureter can occur in up to 1% of colorectal surgical patients, and is associated with increased rates of urinary tract infection, renal failure, sepsis, unplanned reoperation, and a median increase in length of hospital stay by 2 days [1]. Ureteric injury can also result in long-term urological sequelae in approximately one third of patients, such as ureteral strictures, chronic urinary fistulae, recurrent urinary tract infections, and chronic hydronephrosis [2].

We describe a laparoscopic anterior resection where there were difficulties visualizing the left ureter, and the use of intraureteral indocyanine green (ICG) unexpectedly identified a duplicated ureter as well as duplicated renal pelvices. Unbeknownst to most surgeons, the rate of duplicated ureters is up to 6%, as demonstrated by cadaveric studies [3]. In this case, ICG was used not because anatomic anomalies were suspected, but rather due to difficulties in ureteric visualization. This case and literature review thus may demonstrate that novel techniques in ureteric identification such as ICG may reduce the risk posed by anatomic variants in contributing to ureteric injury.

## Case report

**Figure 1:**
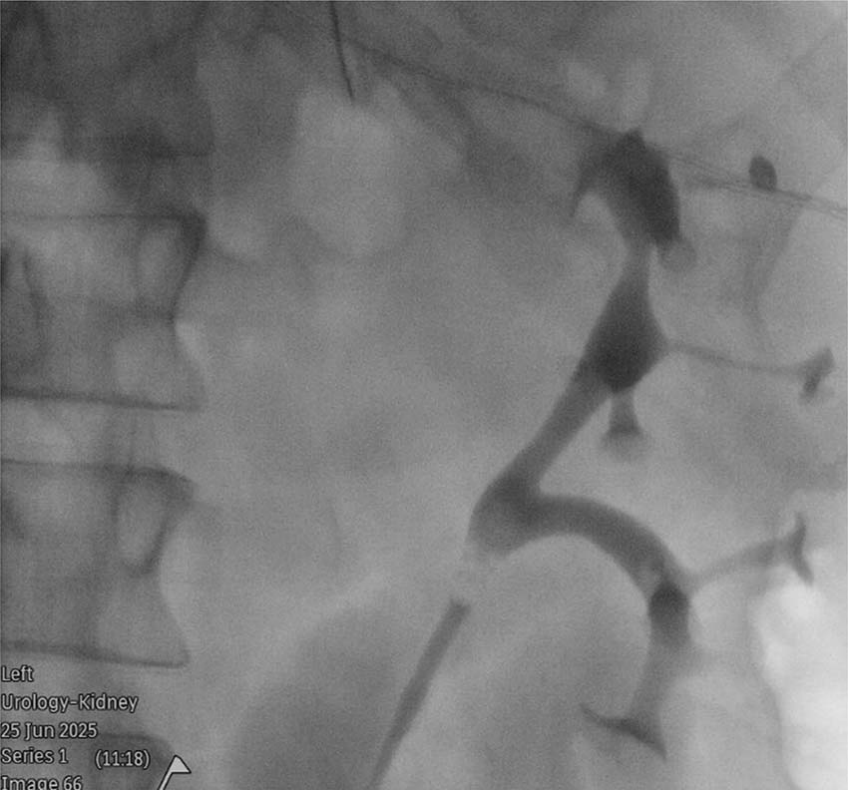
Retrograde urethrogram under fluoroscopic guidance, demonstrating a partially duplicated collecting system, with two renal pelvices converging into a single intact ureter.

**Figure 2:**
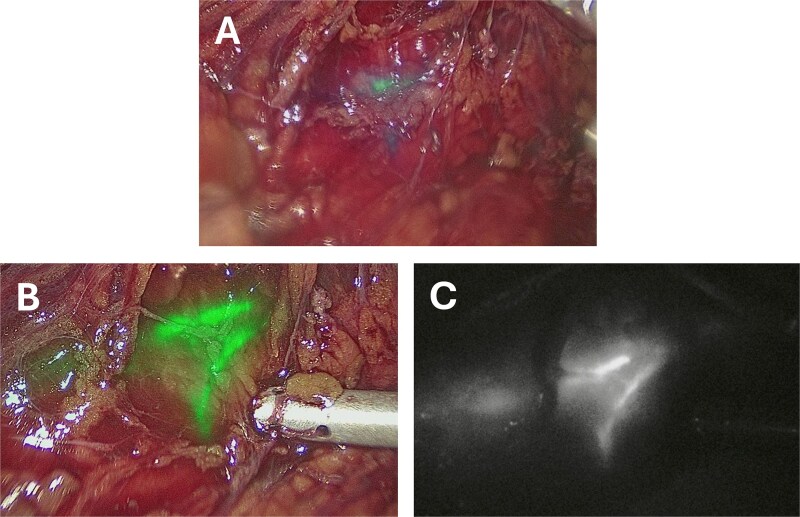
Intraoperative images taken after instillation of 12.5 mg (5 ml) ICG into a left ureteric stent taken using a Storz Opal1® 4 K-NIR/ICG laparoscopic camera. (A) Taken approximately 30 seconds after ICG administration. (B) Taken approximately 5 minutes after administration using the overlay visualization mode. (C) Taken approximately 5 minutes after administration using the monochromatic visualization mode.

A 54-year-old man (Mr MP) presented to the colorectal clinic with recurrent sigmoid diverticulitis, on a background of Crohn’s disease, renal cell carcinoma (managed with right nephrectomy), and obesity (body mass index 32). A laparoscopic anterior resection was performed to remove the most affected segment of bowel. Surgery was difficult due to dense adhesions from diverticulitis, previous surgery, and the patient’s obesity. Despite over an hour of careful dissection, attempts to visualize the left ureter were unsuccessful, and a retrograde urethrogram under fluoroscopic guidance was required. This demonstrated a partially duplicated collecting system, with two renal pelvices converging into a single intact ureter which entered the bladder at a single ureteric orifice (Fig. 1). A ureteric stent was placed, however the left ureter still could not be visualized. As such, the decision was made to use intraureteral ICG to assist with identification. A 25 mg vial of ICG was diluted with 10 ml of sterile water, and a total of 12.5 mg (5 ml) of ICG was injected. This was detected laparoscopically using a Storz Opal1® 4 K-NIR/ICG camera using the Overlay and Monochromatic visualization modes (Fig. 2). This method revealed two distinct tubular fluorescent structures representing duplicated ureters, which were not clearly visible on the retrograde urethrogram. Both branches of the bifid ureter were narrow in caliber, which was the reason for difficulties in its identification. With ICG, the duplicated ureter was traced to its distal lateral location and protected. There were no adverse events from ICG administration. The ureteric stent was removed immediately post-operatively. Mr. MP experienced transient mild hematuria post-stent removal. Mr. MP otherwise made an uncomplicated postoperative recovery, and was discharged at Day 5 post-procedure.

## Discussion

**Figure 3:**
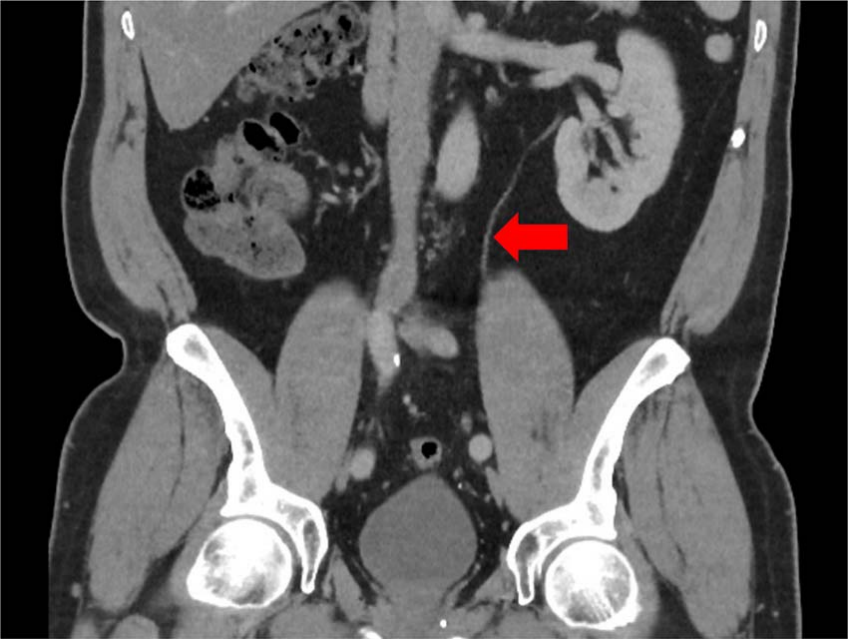
CT scan taken 1 year pre-operatively during a prior episode of acute sigmoid diverticulitis, showing a single ureter (arrow) descending from the left kidney, which entered the bladder at a single ureteric orifice.

Duplicated ureters originate from two non-communicating pyelocaliceal systems, draining into the urinary bladder either at a single ureteric orifice (bifid ureter, incomplete duplication) or by two separate ureteric orifices (double ureter, complete duplication) [3]. This variant occurs due to the formation of multiple or divided ureteric buds from the same mesonephric duct during development [3]. Most cases are found incidentally through imaging such as computed tomography (CT) urograms or intravenous pyelograms [4], through post-mortem studies [3], or occasionally intra-operatively [5, 6]. Less commonly, patients can present with recurrent urinary infections or genitourinary obstruction [3]. In the described case, retrospective review of Mr. MP’s preoperative CT scans with two senior radiologists failed to identify the duplicated ureters (Fig. 3), which may potentially demonstrate the role of ICG as a routine step for ureteric identification in colorectal surgery.

Case reports have described iatrogenic ureteric injury due to a previously unrecognized duplicated collecting system [7, 8], which supports an increased risk of injury in patients with variant genitourinary anatomy. Further, one study analysed cadaveric dissections and CT urograms of 10 cases with bifid ureter, and found that the two ureters were typically unequal in caliber, with the smaller branch presumably draining a smaller area of renal parenchyma [9]. The presence of narrow caliber duplicated ureters, as seen in the present case, could also feasibly increase the risk of iatrogenic injury.

Techniques aiding intraoperative identification of the ureters include placement of a lighted ureteric stent or near-infrared fluorescent ureteral catheter, and anterograde (intravenous) or retrograde (intraureteral) administration of fluorescent dye such as ICG or methylene blue [10]. In this case, we utilized intraureteral ICG administered via a temporary stent, which facilitated rapid intraoperative identification of the bifid ureter within minutes of administration by binding to and reversibly staining the ureteral epithelium. Although ICG may rarely cause anaphylaxis when administered intravenously, it does not typically cause adverse events when administered intraureterally [11, 12]. Transient hematuria was reported in up to 50% of cases of stent-assisted ICG administration in a systematic review including 142 patients [11], and although one of these cases experienced prostate bleeding and one developed ureteral stenosis, neither of these complications were observed in our patient post-operatively. This emerging evidence suggests that intraureteral ICG is a safe and effective method of intraoperative visualization of the ureters.

## Conclusions

Ureteric injury is a feared complication of colorectal surgery. Variant ureteric anatomy is common but is not widely known. The risk of ureteric injury may be greater in patients with a duplicated ureters. Techniques to aid intraoperative identification of the ureters, such as administration of ICG, may be considered as part of an armamentarium of new technology to reduce the risk of complications in colorectal surgery, especially where unfavorable anatomy or dense inflammatory adhesions prevent adequate visualization and safe dissection.

## References

[1] McClelland PH, Liu T, Johnson RP et al. Iatrogenic urinary injuries in colorectal surgery: outcomes and risk factors from a nationwide cohort. Tech Coloproctology 2024;28:137. 10.1007/s10151-024-03008-z 39361072

[2] Sérénon V, Rouanet P, Charleux-Muller D et al. Iatrogenic ureteral injury during colorectal surgery has a significant impact on patient outcomes: a French multicentric retrospective cohort study. Colorectal Dis 2023;25:1433–45. 10.1111/codi.16630 37254657

[3] Arumugam S, Subbiah NK, Mariappan SA. Double ureter: incidence, types, and its applied significance-a cadaveric study. Cureus 2020;12:e7760. 10.7759/cureus.7760 32455077 PMC7243094

[4] Prakash RT, Venkatiah J, Bhardwaj AK et al. Double ureter and duplex system: a cadaver and radiological study. Urol J 2011;8:145–8. 21656475

[5] Alberts VP, Minnee RC, van Donselaar-van der Pant KAMI et al. Duplicated ureters and renal transplantation: a case-control study and review of the literature. Transplant Proc 2013;45:3239–44. 24182792 10.1016/j.transproceed.2013.06.012

[6] Lau SYC, Mirmilstein G, Wardill D. Enhanced localization of duplicated ureters in robotic Hartmann’s reversal using indocyanine green. ANZ J Surg 2020;90:634–5. 10.1111/ans.15286 31087477

[7] Tardu A, Kayaalp C, Ertugrul I et al. Identification of ureter during colorectal surgery cannot always avoid ureteral injury: duplicated collecting system. Am Surg 2015;81:369. 26672568

[8] Hakim JI, Basu A, Luchey A et al. Treatment of the duplicated ureter injured intraoperatively, application of kidney transplant techniques to the urology reconstruction setting: case report and review of the literature. Curr Urol 2010;4:107–9.

[9] Vărgău M, Iliescu DM, Ionescu C et al. Morphological aspects of the bifid ureter (in Y). ARS Medica Tomitana 2013;19:223–8.

[10] Brollo PP, Puggioni A, Tumminelli F et al. Preventing iatrogenic ureteral injury in colorectal surgery: a comprehensive and systematic review of the last 2 decades of literature and future perspectives. Surg Today 2024;54:291–309. 36593285 10.1007/s00595-022-02639-9

[11] Garoufalia Z, Wexner SD. Ureter identification utilizing indocyanine green (ICG) imaging in colorectal surgery: a systematic review of the literature. Mini-Invasive Surg 2022;6:6. 10.20517/2574-1225.2022.60

[12] Keller NB, Stapler SJM, Shanker BA et al. Anaphylactic shock to intravenous indocyanine green during a robotic right colectomy. Am Surg 2023;89:6407–9. 37840264 10.1177/00031348231206584

